# Early-life undernutrition in the great Chinese famine and the risk of early natural menopause: a retrospective cohort study in Western China

**DOI:** 10.3389/fnut.2024.1432707

**Published:** 2024-11-01

**Authors:** Xiaoyang Xu, Yong Zhang, Xiaoya Qi

**Affiliations:** ^1^Health Medicine Center, The Second Hospital Affiliated to Chongqing Medical University, Chongqing, China; ^2^School of Public Health, Chongqing Medical University, Chongqing, China

**Keywords:** early menopause, famine, Chinese woman, retrospective cohort, developmental origins

## Abstract

**Purpose:**

Early age of menopause may increase the risk of fracture, cardiovascular diseases, and all-cause mortality. This study aimed to investigate the relation between early-life undernutrition in the Great Chinese Famine and the risk of early natural menopause.

**Methods:**

A famine exposure retrospective cohort was established during 2017–2018. Postmenopausal women who were born on 01 October 1956–30 September 1964 and came to the hospital for routine health examinations were candidates for the study. Famine time was defined from 1 January 1959 to 31 December 1961. Three types of early-life famine exposure status were determined by the participant’s date of birth. Natural menopause age below 45 was defined as early menopause. The association between early-life famine exposure status and the risk of early natural menopause was confirmed by multiple logistic regression.

**Result:**

A total of 3,337 participants born around famine were included in this study. The prevalence of early menopause was 13.1, 10.0, and 8.3% for those born before, during, and after the famine, respectively. The multiple logistic regression showed that women born before famine significantly increased the risk of early menopause compared to non-exposure (born after famine) (the fully adjusted OR = 1.463, 95%CI = 1.049–2.042). The fetal famine exposure did not significantly increase the risk of early menopause (the fully adjusted OR = 1.244, 95%CI = 0.878–1.764).

**Conclusion:**

Long-term early childhood famine exposure, which caused chronic undernutrition at young ages, increased the risk of early menopause. Early lifetime undernutrition can be recognized as an adverse factor in female reproductive development and aging. This cohort study further confirmed the hypothesis of developmental origins of health and disease from the aspect of women’s reproductive health. Further mechanism study is warranted.

## 1 Introduction

As human life expectancy increases, more and more time in a woman’s life is spent after menopause, and it is estimated that 1.2 billion women will be menopausal by the year 2030 in the world (1, 2). Menopause is the result of the process of ovarian decline to failure. During this process, the female body has various changes, including changes in the menstrual cycle and hormone levels, and can even cause mood disorders such as anxiety and depression (3, 4). Early age of menopause or early ovarian failure may increase the risk of fracture (5), hypertension (6), CVD (7), stroke, atherosclerosis, and all-cause mortality (8). Studies showed that many factors could affect the age of menopause, such as regional, environmental, and nutrition (9).

Famine is a severe food shortage disaster and can cause severe chronic or acute undernutrition in a population, especially pregnant women and children (10). According to the Developmental Origins of Health and Disease (DoHaD) theory, various diseases and conditions in adults are at least partly due to early-life (fertilization, embryonic, fetal, and neonatal and young childhood stages) harmful exposures while tissues and organs are developing (11). Many studies have confirmed the effects of famine exposure in early life on adult hypertension, hyperglycemia, dyslipidemia, hyperuricemia, cognition, and physical function (12–15). However, studies have been insufficient and inconsistent on the relationship between famine and menopause. A systematic review in 2017 only identified three studies about famine and menopause (16). For example, Elias et al. (17) found caloric restriction (exposure to the 1944–1945 Dutch famine) in early childhood (2–6 years of age) can significantly decrease the age of natural menopause. Wang et al. found that fetal, not childhood, exposure to famine was associated with an increased risk of early menopause (18). On the contrary, Lewington et al. found no relationship between famine exposure and menopause (19). Therefore, there is a need to provide more robust evidence about the effects of famine exposure in early life on women’s menopause age which may help us understand the mechanism of reproductive aging.

## 2 Methods

### 2.1 Study population

In this retrospective cohort study, postmenopausal women who underwent physical examinations from 1 October 2017 to 31 December 2018, at the Health Management Center of the Second Affiliated Hospital of Chongqing Medical University, China, were enrolled for this cohort. Those born around the year of the Great Chinese Famine (1959–1961), that is, from 01 October 1956 to 30 September 1964 in this study, were included. Women with any medical history before menopause, such as sex hormone or surgical treatment, which may change the time of menopause, were excluded.

Approval of this study from the Ethics Committee of the Second Affiliated Hospital of Chongqing Medical University was obtained (No. 2021-96-1). Because the de-identified medical record was used, no informed consent was required from the participants.

### 2.2 Definitions of famine exposures

The exact dates of the Chinese famine’s start and end needed to be clarified and varied across regions. Based on a study that confirmed the famine time with a large-scale census population in Chongqing city (20), we arbitrarily set the start point of famine as 1 January 1959 and the endpoint as 31 December 1961. Participants were then categorized into three groups according to their birthdays as follows:

Born before famine (childhood exposure): born between 1 October 1956 and 31 December 1958 and experienced famine in their early childhood stage;

Born during famine (fetal exposure): who was born between 1 January 1959 and 31 December 1961 and experienced famine in their fetal stage and infant stage;

Born after famine (non-exposure): born between 1 January 1962 and 30 September 1964 and free of famine exposure.

### 2.3 Definition of early natural menopause

Menopause was defined as a lack of menstrual bleeding for at least 12 months. Early menopause was defined as the natural menopause age below 45 without any identified medical reasons.

### 2.4 Menstrual history and childbearing history

A gynecologist routinely collected menstrual history and childbearing history in detail when interviewing participants during voluntary gynecological health consulting. The number of gravidities referred to the number of pregnancies regardless of their outcome, and the number of living children referred to the total number of children the participants had. Number of abortions refers to the number including all miscarriages, medical abortions, or surgical abortions during pregnancy.

### 2.5 Physical examination

Height and weight were measured after participants took off their shoes and coats. BMI was calculated as weight (kg)/height (m)2.

### 2.6 Data analysis

De-identified data from the physical examination records were inquired, extracted, and checked. Continuous variables were expressed as mean ± standard deviation, and category variables were expressed as numbers and percentages. The comparisons among different groups were conducted by *t*-test, analysis of variance (ANOVA), or chi-squared test. The risk of early menopausal age caused by famine exposure was analyzed by multivariable logistic regression, and potential confounders such as weight, height, BMI, menarche age, number of gravidities, number of living children, and number of abortions were included in multivariable logistical regression to eliminate their influence on early menopause. All data processing and analysis used the statistical software SPSS 24.0.

## 3 Results

### 3.1 Famine exposure cohort

During the enrolling period of October 2017–December 2018, 7,278 menopausal women born between 1956 and 1964 and who visited the hospital for health examination entered this famine exposure cohort. 3,337 (45.8%) of them received gynecological health consulting and provided information on menopause age. Based on the first four numbers of their identification numbers, 3,176 (95.18%) participants were identified as being born in the Chongqing area and were included in the analysis. Included participants were grouped according to their birth date into three categories, that is, childhood exposure, fetal exposure, and non-exposure. As shown in Table 1, the participants included were younger than those who were excluded because a higher portion of non-exposure was included.

**Table 1:** Comparison of included and excluded participants in the famine exposure cohort.

Birth years	Famine exposure	Included participants (*n* = 3,337)	Excluded participants (*n* = 3,941)	^#^*p*-value
Before famine (01 October 1956 ~ 31 December 1958)	Childhood exposure	663 (37.8%)	1,093 (62.2%)	>0.001
During famine (01 January 1959 ~ 31 December 1961)	Fetal exposure	667 (42.2%)	914 (57.8%)	
After famine (01 January 1962 ~ 30 September 1964)	Non-exposure	2007 (50.9%)	1934 (49.1%)	

^#Chi-square test.^

### 3.2 Characteristics of included famine exposure cohort

Those who were born before (childhood exposure) and during famine (fetal exposure) were shorter and lighter, earlier at menopause, and had less gravidity and abortion times than those who were born after famine (non-exposure) (*p* < 0.05), and at the same time, a trend also exists among the three groups (P_trend_ < 0.05). These women were not significantly different in BMI, menarche age, or living children numbers (*p* > 0.05) (see Table 2).

**Table 2:** Characteristics of included participants by famine exposure cohorts (mean ± S.D).

Variables	Childhood exposure (*n* = 663)	Fetal exposure (*n* = 667)	Non-exposure (*n* = 2007)	*p*-trend
Birthday range	10/01/1956 ~ 12/31/1958	01/01/1959 ~ 12/31/1961	01/01/1962 ~ 09/30/1964	**-**
Weight (kg)	56.7 ± 8.1*	56.6 ± 8.0*	57.8 ± 7.8	0.001
Height (cm)	155.2 ± 5.7*	155.4 ± 5.7*	156.5 ± 5.4	<0.001
BMI	23.5 ± 3.1	23.5 ± 3.0	23.6 ± 3.1	0.517
Menarche age	14.0 ± 1.4	13.9 ± 1.4	13.9 ± 1.3	0.282
Menopause age	49.6 ± 3.9*^#^	50.0 ± 3.5	50.1 ± 3.3	0.006
Number of Gravidity	2.5 ± 1.5*	2.6 ± 1.5	2.7 ± 1.6	0.005
Number of Living children	1.3 ± 0.7	1.2 ± 0.6	1.3 ± 0.7	0.737
Number of Abortion	1.3 ± 1.3*	1.4 ± 1.4	1.5 ± 1.4	0.001

^*^p < 0.05 when compared with non-exposure; ^#^compared with feta-exposure. Analysis of variance (ANOVA) for trend analysis. Least significant difference (LSD) for paired group comparison.

### 3.3 The prevalence of early natural menopause age by famine exposure

The prevalence of early menopause was 12.8, 10.0, and 8.3% in groups born before (childhood exposure), during (fetal exposure), and after the famine (non-exposure), respectively. Moreover, the prevalence in the group born before famine is significantly higher than that in those born after famine (*p* < 0.05) (see Table 3). No significant difference in early menopause prevalence was found between the other two groups (*p* > 0.05). Nevertheless, there is a significant decreasing trend for early menopause prevalence in those born before, during, and after the famine (*P*_trend_ < 0.05).

**Table 3:** Prevalence of early menopause by famine exposure cohorts.

Famine exposure	Prevalence of early menopause	95%CI of prevalence	*p*-chi-square for 3 groups	*p*-trend
Childhood exposure	12.8% (85/578)*	10.4–15.8%	0.002	0.001
Fetal exposure	10.0% (67/600)	7.7–12.4%		
Non-exposure	8.3% (166/1841)	8.5–10.5%		

^*^p < 0.05 when compared with non-exposure; chi-square test for overall and paired group comparing and trend analysis.

### 3.4 The multiple logistic regression of famine exposure and early natural menopause

The logistic regression analysis showed that women born before famine (childhood exposure) have a higher risk of early menopause (the fully adjusted OR = 1.463, 95%CI, 1.049–2.042) than those born after the famine (non-exposure). Those born during famine (fetal exposure) also had a higher risk but not significant when referring to those born after famine group (Table 4).

**Table 4:** Multiple logistic regression of famine exposure on nearly natural menopause.

Models	OR (95% CI) of early menopause		
	Childhood exposure (*n* = 663)	Fetal exposure (*n* = 667)	Non-exposure (*n* = 2007)
Model1	1.521 (1.094, 2.114)	1.262 (0.862, 1.785)	ref
Model2	1.486 (1.067, 2.071)	1.244 (0.878, 1.763)	ref
Model3	1.463 (1.049, 2.042)	1.244 (0.878, 1.764)	ref

Model 1, unadjusted; model 2, adjusted by weight, height, and BMI; model 3, model 2 plus menarche age, number of gravidities, number of living children, and number of abortions.

## 4 Discussion

This retrospective famine exposure cohort study was established to explore the relationships between early-life famine exposure and menopause age. The main results showed that early childhood famine exposure (born before famine) had significantly lower menopause age (49.6 vs. 50.1) and higher risk of early menopause age (OR = 1.463) than non-famine exposure (born after famine). However, those born during the famine (mainly fetal exposure) showed the same trend of changes in menopause age without significance compared to the non-exposure group (born after famine). This may indicate that early childhood stages are more sensitive to famine than the fetus and infant stages because the maternal body may buffer the effects of adverse events on the fetus and infants (21). However, this buffer function of the maternal body was rarely noticed in famine studies in current studies. Notwithstanding, our study in some aspects further confirmed the Developmental Origins of Health and Disease (DoHaD) theory (11), that is, early-life exposure to famine could change the reproductive aging process and lead to early menopause.

In detail, our results were not the same as the study conducted by Wang et al. (18). Although both studies were performed in China and had the same aged population, Wang’s study showed that fetal exposure (born during famine) had more potent effects than childhood exposure (born before famine), which was contrary to our results. In Wang’s study, the overall prevalence of early menopause was 6.4% (non-exposure), 7.3% (fetal exposure), and 4.8% (childhood exposure), which were smaller than our results. This may be because Wang’s study was community-based while this study was hospital-based, and medical records are more reliable. Furthermore, a study (22) by Yarde on Dutch famine showed that women with famine exposure in fetuses were more likely to reach menopause at any age at the time of investigation than age-paralleled non-exposure control, which may mean that women with fetus exposure to famine may experience menopause at a younger age. However, the diagnosis of early menopause was not introduced and analyzed by Yarde. Furthermore, the famine of the Dutch lasted only for 6 months, which may be the source of inconsistency. However, our results were consistent with another Dutch famine study by Elias et al. (17), which showed severe early childhood (2–6 years of age) exposure to famine had significant effects on menopause with 1.83 years earlier than unexposed women. Still, early menopause was not diagnosed in this study. Those inconsistent parts among studies in this field may be due to the differences in study methods used, such as studying setting, the sample size, the data source, and exposure definition and risk estimates, as proposed in a systematic review on this topic (16).

Many factors can lead to early menopause, such as premature ovarian insufficiency, smoking, early menarche, shorter menstrual cycles, and stress (23). Regarding the mechanisms of early-life famine exposure on menopause, chronic or acute malnutrition due to calorie restriction or nutrient deficiency in early life can cause pre-or post-natal development restriction, for example, manifested as low birthweight, which then leads to perimenopausal disorders (24), premature aging, and shortened lifespan (25–27). Aging of the reproductive system precedes the body (28). Famine exposure in early life could reduce the number of ovarian germ cells and cause ovarian reserve to decrease, resulting in earlier menopause. Animal experiments by Aiken et al. (29) found that in uterine nutrition-deficient rats, oxidative stress levels increased, the length of the fallopian tube and ovaries telomere decreased faster, cells aged faster, and telomerase activity decreased, leading to ovarian insufficiency (30). Thus, malnutrition in the early-life stage may advance the menopause age, which shortens the reproductive period in women.

It is worth noting that menarche ages were not influenced by earlier life famine exposure in this study and also the study conducted by Wang (18). Nevertheless, menarche age delayed by famine was observed in other studies, especially in the late childhood exposure group (31). As menarche age is negatively related to menopause age (32), this may implicate that late childhood food shortage and malnutrition might have a more profound influence on menopause age, as supported by the Kalichman study, which assessed the effects of famine exposure during puberty and found that menopause occurred 1.7 years earlier (33). In addition to the buffer function of the maternal body (21), some after-birth adverse events may attenuate or distort the influence of fetal exposure and this may partly explain why fetal exposure (born during famine) had insignificant effects on menarche age and menopause age in our study.

Our study has some strengths worthy of mention. First, it was a large sample size cohort study with good generalization to the whole West China population. Second, the participants were enrolled through health examinations and were relatively healthy, which may exclude other medical conditions at menopause. Third, data and information come from medical records, which are more reliable than other ways. However, some limitations were inevitable. For example, we cannot include the person who died when conducting this study. This may underestimate the effects of premature exposure to famine. In addition, this study did not offer factors such as smoking, drinking, physical activity, oral contraceptives, fertility rate, mortality rate, and birth characteristics. Therefore, we cannot exclude the influences of those confounders. Finally, all participants came from one hospital and chose free gynecological consultation for their health examination report. They may have some standard features that can cause selection biases. The multi-center study is warranted in the future.

## 5 Conclusion

Long-term early childhood famine exposure, which caused chronic undernutrition at young ages, increased the risk of early menopause. Early lifetime undernutrition can be recognized as an adverse factor in female reproductive development and aging. This cohort study further confirmed the hypothesis of developmental origins of health and disease from the aspect of women’s reproductive health. Further mechanism study is warranted.

## Data Availability

The raw data supporting the conclusions of this article will be made available by the authors, without undue reservation.
